# Addressing racial and ethnic disparities in US liver cancer care

**DOI:** 10.1097/HC9.0000000000000190

**Published:** 2023-06-22

**Authors:** Nicole J. Kim, Anne Cravero, Trang VoPham, Philip Vutien, Rotonya Carr, Rachel B. Issaka, Janet Johnston, Brian McMahon, Jorge Mera, George N. Ioannou

**Affiliations:** 1 Division of Gastroenterology, Department of Medicine, University of Washington, Seattle, Washington, USA; 2 Department of Medicine, University of Washington, Seattle, Washington, USA; 3 Epidemiology Program, Public Health Sciences Division, Fred Hutchinson Cancer Center, Seattle, Washington, USA; 4 Department of Epidemiology, University of Washington School of Public Health, Seattle, Washington, USA; 5 Clinical Research Division, Fred Hutchinson Cancer Center, Seattle, Washington, USA; 6 Liver Disease and Hepatitis Program, Alaska Native Tribal Health Consortium, Anchorage, Alaska; 7 Cherokee Nation Health Services, Tahlequah, Oklahoma; 8 Division of Gastroenterology, Department of Medicine, Veterans Affairs Puget Sound Health Care System, Seattle, Washington, USA

## Abstract

HCC, the most common form of primary liver cancer, is the fastest rising cause of cancer-related death in the United States. HCC disproportionately affects racial and ethnic minorities in the United States. A practical framework is needed to organize the complex patient, provider, health system, and societal factors that drive these racial and ethnic disparities. In this narrative review, we adapted and applied the National Institute on Minority Health and Health Disparities (NIMHD) Research Framework to the HCC care continuum, as a step toward better understanding and addressing existing HCC-related disparities. We first summarize the literature on HCC-related disparities by race and ethnicity organized by the framework’s 5 domains (biological, behavioral, physical/built environment, sociocultural environment, and health care system) and 4 levels (individual, interpersonal, community, and societal) of influence. We then offer strategies to guide future research initiatives toward promotion of health equity in HCC care. Clinicians and researchers may help mitigate further inequities and better address racial and ethnic disparities in HCC care by prioritizing the following in HCC research: (1) increasing racial and ethnic minority representation, (2) collecting and reporting HCC-related data by racial and ethnic subgroups, (3) assessing the patient experience of HCC care by race and ethnicity, and (4) evaluating HCC-specific social determinants of health by race and ethnicity. These 4 priorities will help inform the development of future programs and interventions that are tailored to the unique experiences of each racial and ethnic group.

## Graphical abstract

**Figure:**
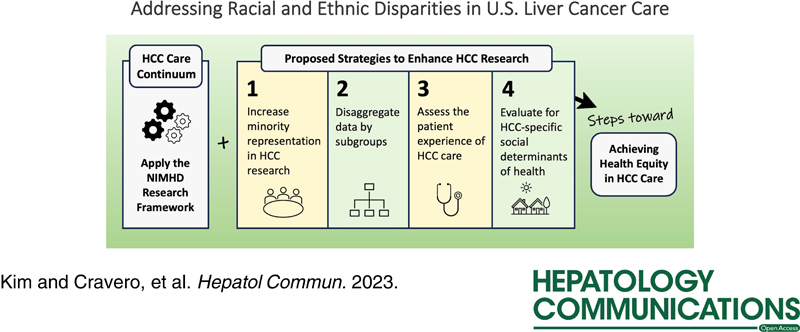

## INTRODUCTION

HCC, the most common form of primary liver cancer, is the fastest growing cause of cancer-related death in the United States^1^ However, the burden of HCC in the United States is not experienced equally. HCC-related disparities by race and ethnicity exist along the entire HCC care continuum, from early identification of cirrhosis to HCC-related mortality. These disparities are thought to be multifactorial in origin and are linked to patient, provider, and health system factors. Such factors may include downstream and midstream social determinants of health,^2–4^ which include but are not limited to individual health literacy, insurance coverage, and neighborhood poverty. In addition, more upstream factors,^2–4^ such as systemic racism and discriminatory US policies (eg, redlining to exclude Black Americans, immigrants, and working-class people from securing home or business mortgage loans^5–7^) disproportionately affect racial and ethnic minorities, further exacerbating disparities in care.^8^

Evaluating HCC disparities by race and ethnicity has its limitations (e.g., race and ethnicity are social constructs, often self-reported, and do not fully account for the genetic and cultural diversity of a racial or ethnic group^9,10^), but it can provide an opportunity for clinicians, researchers, and Tribal Health Organizations to advocate for interventions and policies that may address some of the existing inequities in liver cancer care. In this narrative review, we therefore present an adapted version of the National Institute on Minority Health and Health Disparities (NIMHD) Research Framework^11^ to summarize the potential drivers of existing disparities in HCC care by race and ethnicity. We then provide potential strategies to guide future research initiatives to address knowledge gaps, mitigate further inequities, and more effectively address existing disparities along the HCC care continuum.

### Applying the NIMHD research framework to HCC care

The National Cancer Institute’s Translational Liver Cancer (TLC) consortium recently proposed a conceptual model to highlight the steps along the HCC screening process, including risk assessment, screening initiation, result follow-up, diagnostic evaluation, and treatment evaluation.^12^ We combined the TLC screening continuum with the NIMHD Research Framework^11^ to develop a more comprehensive framework that includes an expanded HCC care continuum (Table 1), which incorporates HCC risk identification and incidence, receipt of HCC surveillance, early detection, treatment, and mortality, to identify various factors that might contribute to the differences in HCC outcomes by race and ethnicity.

**TABLE 1:**
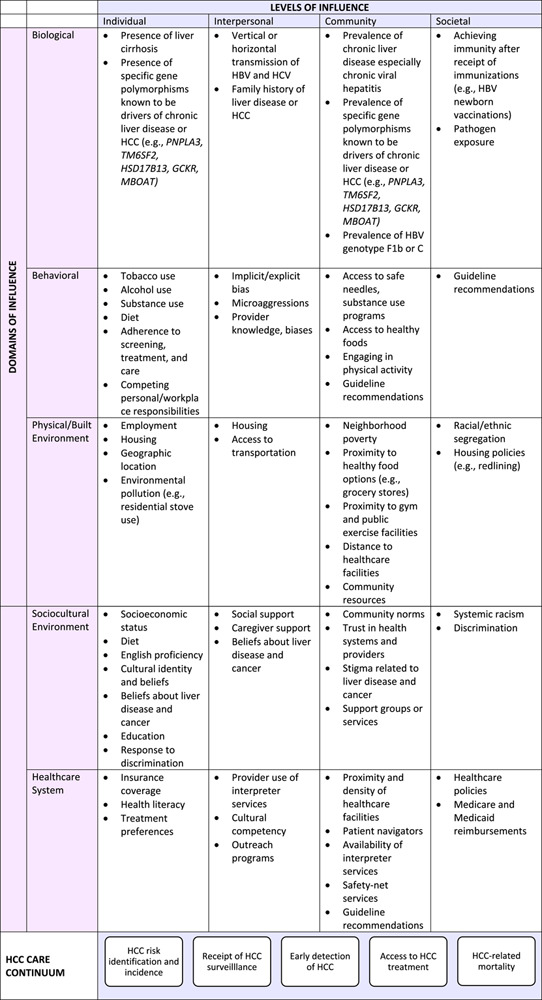
Proposed framework for assessing HCC-related disparities by race and ethnicity

Abbreviations: NIMHD, National Institute on Minority Health and Health Disparities.

Adapted from the NIMHD Research Framework.^11^

The NIMHD Research Framework^11^ has been successfully used to promote research on disparities in other settings, including in end-of-life care among Black Americans^13^ and the inclusion of Arab/Middle East/North African communities in disparity research.^14^ However, to our knowledge, it has never been applied in liver cancer care. As shown in Table 1, the framework can be used to systematically organize the patient, provider, health system, and societal factors that drive racial and ethnic disparities in HCC care. The framework includes 5 domains (biological, behavioral, physical/built environment, sociocultural environment, and health care system) and 4 levels (individual, interpersonal, community, and societal) of influence. Each factor falls into 1 domain and 1 level of influence and can be specifically targeted for an intervention. Alternatively, multiple factors across domains and/or levels of influence may be addressed simultaneously using multilevel interventions. As an example, “achieving immunity after receipt of vaccinations” would fall within the biological domain and societal level of influence, while “cultural identity” would fall within the sociocultural environment domain and individual level of influence.^11^ In this review, we combined all factors across racial and ethnic groups into 1 framework for ease of visualization. However, this framework should be further tailored to the experiences of specific racial and ethnic minority groups for it to be most effective. Our goal is for this framework to be used in public health, clinical, and research settings to guide future programs and research initiatives that can effectively address liver cancer disparities.

### Definitions used in this review

This review focuses on the racial and ethnic minority groups commonly identified by studies in the United States—African American or Black, American Indian and Alaska Native (AI/AN), Asian American or Asian and Pacific Islander (API), and Hispanic or Latinx.^9^ We recognize that many studies combine the Asian American, Native Hawaiian, and Pacific Islander populations, which may limit the interpretation of findings. As HCC is the most common form of primary liver cancer, the terms “liver cancer” and “HCC” are also used interchangeably.

### HCC-related disparities by race and ethnicity

To illustrate how the NIMHD framework can be used to describe the potential drivers of HCC-related disparities, we present model examples for each step of the HCC care pathway by framework domain (eg, the impact of the biological domain on HCC incidence) and level of influence. However, we recognize that factors from multiple domains and levels of influence might affect the same step in the HCC care continuum (eg, behavioral and health care system factors, or interpersonal and community-level factors may affect receipt of HCC surveillance). As a result, we have also included a table highlighting these associations by race and ethnicity (Table 2).

**TABLE 2:** Selected factors associated with the HCC care continuum by domain of influence and racial and ethnic minority group (reference group: White)

	Factors shared across racial and ethnic minority groups	Black or African American	American Indian/Alaska Native	Asian American or Asian and Pacific Islander	Hispanic or Latinx
HCC risk identification and incidence	**Biological** Access to (newborn HBV) vaccinations Family history of liver disease or HCC Underlying chronic liver disease **Behavioral** ^a^ Alcohol use Diet Obesity Physical activity **Physical/built environment** Geographic location of residence Neighborhood socioeconomic status **Health care system** Location of HCC diagnosis (eg, emergency department)	**Biological** High HCV prevalence	**Biological** HBV genotype F1b or genotype C	**Biological** High HBV prevalence Emigration from countries with high HBV endemicity Vertical HBV transmission **Sociocultural environment** Birthplace (US vs. foreign-born) HBV-related internalized and social stigma	**Biological** HBV genotype F1b PNPLA3 gene polymorphism Sociocultural environment Birthplace (US vs. foreign-born)
Receipt of HCC surveillance	**Biological** Presence of advanced fibrosis or cirrhosis Family history of liver disease, HBV, or HCC **Behavioral** Patient awareness of HCC risk Provider knowledge of clinical guidelines Provider perceptions of HCC screening Provider implicit racial/ethnic bias **Physical/built environment** Proximity to screening facility **Sociocultural environment** Beliefs about liver disease and cancer **Health care system** Ease of scheduling screening Cost of screening	**Behavioral** Lower rates of referral to specialty clinic Provider notification for HCC screening **Sociocultural environment** English proficiency **Health care system** Health insurance Patient navigators	**Sociocultural environment** English proficiency **Health care system** Proximity to Indian Health Service or Tribal health programs	**Sociocultural environment** English proficiency **Health care system** Patient navigators	**Behavioral** Lower rates of referral to specialty clinic Provider notification for HCC screening **Sociocultural environment** English proficiency **Health care system** Patient navigators
Early detection of HCC	**Behavioral** Receipt of HCC screening for those with documented risk factors **Sociocultural environment** Beliefs about liver disease and cancer **Health care system** Location of diagnosis (eg, emergency department)	**Behavioral** Provider clinical reminders for HCC screening **Physical/built environment** Counties with high social vulnerability index scores Sociocultural environment English proficiency **Health care system** Patient navigators	**Sociocultural environment** English proficiency **Health care system** Proximity to Indian Health Service or Tribal health programs	***Sociocultural environment*** English proficiency **Health care system** Patient navigators	***Behavioral*** Provider clinical reminders for HCC screening **Physical/built environment** Counties with high social vulnerability index scores **Sociocultural environment** English proficiency **Health care system** Patient navigators
Access to HCC treatment	**Sociocultural environment** Caregiver support for transplant **Health care system** Proximity to health care facility Referral to liver transplant or cancer center	**Behavioral** Lower rates of HCC detection when curative treatment (hepatic resection, ablation, liver transplant) is possible Lower rates of immunotherapy and palliative care Provider implicit bias Provider notification of diagnosis	**Behavioral** Lower rates of HCC detection when curative treatment (hepatic resection, ablation, liver transplant) is possible **Health care system** Proximity to Indian Health Service or Tribal health programs	**Behavioral** Lower rates of liver transplant Higher rates of hepatic resection and curative therapy Provider implicit bias Provider notification of diagnosis	**Behavioral** Lower rates of HCC detection when curative treatment (hepatic resection, ablation, liver transplant) is possible Lower rates of immunotherapy and palliative care Provider implicit bias Provider notification of diagnosis
HCC-related mortality	**Behavioral** Receipt of HCC screening Receipt of HCC curative treatment **Sociocultural environment** Socioeconomic status **Health care system** Proximity to liver transplant and cancer center	**Behavioral** Provider notification of diagnosis **Physical/built environment** Neighborhood racial segregation **Sociocultural environment** Economic mobility gap **Health care system** Health insurance	**Sociocultural environment** Socioeconomic status **Health care system** Proximity to Indian Health Service or Tribal health programs	**Behavioral** Provider notification of diagnosis **Sociocultural environment** Birthplace (US vs. foreign-born) HBV-related internalized and social stigma **Health care system** Health insurance	**Behavioral** Provider notification of diagnosis Sociocultural environment Birthplace (US vs. foreign-born) **Health care system** Health insurance

a These factors, among many others, are not found exclusively in racial and ethnic minority groups and may also affect the HCC care continuum of White people.

#### HCC risk identification and incidence

- **Impact of the biological domain**

US population–based Surveillance, Epidemiology, and End Results (SEER) data consistently show that AI/AN people have the highest annual HCC incidence rate (15.7/100,000 persons), followed by Hispanic (13.5/100,000 persons), API (12.6/100,000 persons), Black (11.0/100,000 persons), and White (7.1/100,000 persons) people.^15^ Between 2003 and 2011, Hispanic and Asian American people experienced a 35.8% increase and 5.5% decrease in HCC incidence, respectively.^16^ Between 1992 and 2018, AI/AN populations experienced a 4.3% annual increase in HCC incidence.^17^ By 2030, Hispanic and Black persons are projected to have the highest HCC incidence rates.^18^

These differences in HCC incidence may be driven in part by biological factors at the individual level (individual level of influence) (Table 1). For example, the risk of developing HCC is highest among individuals with liver cirrhosis, chronic hepatitis B (HBV) infection, who are males > 40 years and females >50 years of age, and in those with a family history of HCC.^19^

HCC incidence may also vary by race and ethnicity due to biological factors at the community level (community level of influence). The predominant etiology of underlying liver cirrhosis and associated risk of HCC may vary by race and ethnicity due to the high community burden of specific chronic liver diseases (eg, alcohol-associated liver disease, NAFLD, HBV, and HCV) that are present in each minority group. As an example, HCC incidence may be high among Alaska Native people due to the burden of HBV, HCV, and autoimmune hepatitis.^20–23^ The HBV genotype F1b is a strong risk factor for HCC and is found in Southwest Alaska, Spain, Argentina, and Chile.^21,24,25^ HBV genotype C is another risk factor for HCC and is found in Northwest Alaska, Siberia, and several East Asian countries.^21,26^ Among American Indian people, the risk of NAFLD and NASH, a leading cause of cirrhosis,^27,28^ may also vary by Tribal group. For example, the Pima Indian population experiences a disproportionately high rate of type 2 diabetes,^29^ which is a risk factor for NAFLD and NASH, compared with other AI and minority groups. Among Hispanic people, one of the many factors that might contribute to the rising prevalence of NAFLD and NASH, include the *PNPLA3* gene polymorphism, which is more common in this population and associated with greater hepatic fat content, more severe liver inflammation, and higher risk of HCC.^30^ Multiple studies have also shown that Black people experience a higher prevalence of HCV infection,^31^ which is an independent risk factor for HCC in patients with cirrhosis.^32^ Finally, despite advances in HBV awareness, vaccination, and treatment,^33^ Asian American people bear the greatest burden of HBV-related HCC^34^ due to a large proportion of individuals who emigrated from HBV-hyperendemic countries and high rates of vertical transmission.^35^ These findings highlight the importance of promoting clinical, translational, and genomic HCC research to better recognize risk factors for cirrhosis and HCC, specific to each racial and ethnic group. These results can then inform tailored efforts to promote early identification of liver disease, access to treatment if applicable (eg, direct-acting antiviral treatment for HCV infection), and increase community awareness of HCC risk to reduce existing disparities in HCC incidence.

#### Receipt of HCC surveillance

- **Impact of the behavioral domain**
  HCC surveillance with imaging and alpha-fetoprotein testing is recommended every 6 months in patients with cirrhosis or chronic HBV,^19^ but overall rates of HCC surveillance remain suboptimal.^36^ Receipt of surveillance further varies by race and ethnicity. SEER-Medicare claims data showed that 59% of Black and 49% of Hispanic patients (vs. 47% of White patients) received no HCC screening in the 3 years before their HCC diagnosis.^37^ In other studies, Black and Hispanic patients were 60% less likely than White patients to receive consecutive HCC screening tests^38^ and similarly less likely to be referred to specialty clinics,^39^ where HCC screening is more likely to occur than in primary care settings.^40^
  These differences in the receipt of surveillance may stem from multiple factors that impact the patient and provider interaction (interpersonal level of influence). These factors may include implicit racial/ethnic biases^41^ that affect provider behaviors related to HCC screening, provider knowledge of clinical guidelines,^42^ insufficient clinical time to prioritize HCC screening,^42^ and varying provider perceptions of screening modalities.^43^ There is therefore an ongoing need to identify how to implement successful interventions and surveillance programs to optimize patient and provider interactions in the clinical setting and thereby improve HCC surveillance rates. In addition, as novel biomarkers and imaging offer opportunities for individualized and risk-based HCC surveillance, further evaluating the factors that contribute to interpersonal behaviors related to surveillance will be critical to improving adherence to recurrent surveillance events, irrespective of surveillance modality.
- **Impact of the health care system domain**

Many providers already believe practice guidelines are essential to their decision-making process related to ordering HCC surveillance for their patients.^43^ The presence of clear practice guidelines around HCC surveillance (community level of influence) may therefore also impact patient receipt of surveillance. However, current guidelines are not consistent across specialty and primary care associations. For example, while the American Association for the Study of Liver Diseases, which is likely to influence gastroenterology and hepatology providers’ clinical practices, recommends HCC surveillance in at-risk individuals every 6 months,^19^ the US Preventive Services Task Force,^44^ which is likely to influence primary care clinicians’ cancer screening practices, has not yet provided recommendations on HCC surveillance. As minority patients are more likely to receive care in primary care settings^39^ where HCC screening occurs less often than in specialty clinics,^40^ promoting consistent practice guidelines across specialties may help address disparities in the receipt of HCC surveillance by race and ethnicity.

Similarly, prioritizing multidisciplinary research that can better inform evidence-based practice guidelines (community level of influence) may also promote further equity in the receipt of HCC surveillance. For example, NAFLD is now a leading cause of cirrhosis,^27,28^ but how to best implement HCC surveillance in this growing patient population remains unclear (eg, whether to screen patients with cirrhosis only vs. advanced fibrosis and cirrhosis, the limitations in detecting HCC with ultrasound in the setting of comorbid obesity, etc.).^45^ As a recent study found that the rates of NAFLD-related deaths have disproportionately increased among Black and AI/AN people since the pandemic,^46^ investing in research that evaluates the effectiveness and cost-effectiveness of various surveillance modalities in NAFLD may also help reduce disparities in care by standardizing clinical practice, especially among minority patient groups disproportionately affected by NAFLD.

#### Early detection of HCC

- **Impact of the physical/built environment domain**

The physical/built environment refers to the physical space in which individuals live, learn, and work.^47^ This may include home and school environments, neighborhood walkability, and the availability of grocery stores in a community.

Several studies have already shown that HCC stage at diagnosis varies by race and ethnicity.^48–53^ Compared with White patients, Asian American patients are 13% less likely, while Hispanic and Black patients are respectively 20% and 18% more likely, to present with advanced stage HCC.^54,55^ Multiple physical/built environment factors, such as county-level and neighborhood-level characteristics (community level of influence), may contribute to these disparities. As an example, people living in rural and suburban areas (vs. urban areas) are more likely to present with late-stage HCC, not receive HCC treatment, and die owing to HCC.^56^ A greater proportion of Black and Hispanic patients also live in US counties with higher social vulnerability index scores, and individuals from high social vulnerability index counties are more likely to present with larger tumors and receive nonsurgical interventions for HCC.^57^ Similarly, API and Hispanic people were more likely to develop HCC if they lived in low socioeconomic status (SES) (vs. high SES) neighborhoods.^58^ These findings highlight the importance of assessing and collecting both individual and neighborhood-level social determinants to health to better address inequities in HCC care stemming from geographical and built environment differences by race and ethnicity.

#### Receipt of HCC surveillance

- **Impact of the behavioral domain**
  HCC surveillance with imaging and alpha-fetoprotein testing is recommended every 6 months in patients with cirrhosis or chronic HBV,^19^ but overall rates of HCC surveillance remain suboptimal.^36^ Receipt of surveillance further varies by race and ethnicity. SEER-Medicare claims data showed that 59% of Black and 49% of Hispanic patients (vs. 47% of White patients) received no HCC screening in the 3 years before their HCC diagnosis.^37^ In other studies, Black and Hispanic patients were 60% less likely than White patients to receive consecutive HCC screening tests^38^ and similarly less likely to be referred to specialty clinics,^39^ where HCC screening is more likely to occur than in primary care settings.^40^
  These differences in the receipt of surveillance may stem from multiple factors that impact the patient and provider interaction (interpersonal level of influence). These factors may include implicit racial/ethnic biases^41^ that affect provider behaviors related to HCC screening, provider knowledge of clinical guidelines,^42^ insufficient clinical time to prioritize HCC screening,^42^ and varying provider perceptions of screening modalities.^43^ There is therefore an ongoing need to identify how to implement successful interventions and surveillance programs to optimize patient and provider interactions in the clinical setting and thereby improve HCC surveillance rates. In addition, as novel biomarkers and imaging offer opportunities for individualized and risk-based HCC surveillance, further evaluating the factors that contribute to interpersonal behaviors related to surveillance will be critical to improving adherence to recurrent surveillance events, irrespective of surveillance modality.
- **Impact of the health care system domain**
  Many providers already believe practice guidelines are essential to their decision-making process related to ordering HCC surveillance for their patients.^43^ The presence of clear practice guidelines around HCC surveillance (community level of influence) may therefore also impact patient receipt of surveillance. However, current guidelines are not consistent across specialty and primary care associations. For example, while the American Association for the Study of Liver Diseases, which is likely to influence gastroenterology and hepatology providers’ clinical practices, recommends HCC surveillance in at-risk individuals every 6 months,^19^ the US Preventive Services Task Force,^44^ which is likely to influence primary care clinicians’ cancer screening practices, has not yet provided recommendations on HCC surveillance. As minority patients are more likely to receive care in primary care settings^39^ where HCC screening occurs less often than in specialty clinics,^40^ promoting consistent practice guidelines across specialties may help address disparities in the receipt of HCC surveillance by race and ethnicity.
  Similarly, prioritizing multidisciplinary research that can better inform evidence-based practice guidelines (community level of influence) may also promote further equity in the receipt of HCC surveillance. For example, NAFLD is now a leading cause of cirrhosis,^27,28^ but how to best implement HCC surveillance in this growing patient population remains unclear (eg, whether to screen patients with cirrhosis only vs. advanced fibrosis and cirrhosis, the limitations in detecting HCC with ultrasound in the setting of comorbid obesity, etc.).^45^ As a recent study found that the rates of NAFLD-related deaths have disproportionately increased among Black and AI/AN people since the pandemic,^46^ investing in research that evaluates the effectiveness and cost-effectiveness of various surveillance modalities in NAFLD may also help reduce disparities in care by standardizing clinical practice, especially among minority patient groups disproportionately affected by NAFLD.

#### Early detection of HCC

- **Impact of the physical/built environment domain**
  The physical/built environment refers to the physical space in which individuals live, learn, and work.^47^ This may include home and school environments, neighborhood walkability, and the availability of grocery stores in a community.
  Several studies have already shown that HCC stage at diagnosis varies by race and ethnicity.^48–53^ Compared with White patients, Asian American patients are 13% less likely, while Hispanic and Black patients are respectively 20% and 18% more likely, to present with advanced stage HCC.^54,55^ Multiple physical/built environment factors, such as county-level and neighborhood-level characteristics (community level of influence), may contribute to these disparities. As an example, people living in rural and suburban areas (vs. urban areas) are more likely to present with late-stage HCC, not receive HCC treatment, and die owing to HCC.^56^ A greater proportion of Black and Hispanic patients also live in US counties with higher social vulnerability index scores, and individuals from high social vulnerability index counties are more likely to present with larger tumors and receive nonsurgical interventions for HCC.^57^ Similarly, API and Hispanic people were more likely to develop HCC if they lived in low socioeconomic status (SES) (vs. high SES) neighborhoods.^58^ These findings highlight the importance of assessing and collecting both individual and neighborhood-level social determinants to health to better address inequities in HCC care stemming from geographical and built environment differences by race and ethnicity.

#### Access to HCC treatment

- **Impact of the sociocultural domain**
  Access to HCC treatment is closely linked to HCC stage at diagnosis. However, racial minority patients do not receive the same type of HCC treatment even when presenting at similar disease stages. Black and Hispanic patients are not only the least likely of racial minority groups to receive any HCC treatment^59^ but are also less likely to receive curative therapies (eg., hepatic resection and ablation),^50,53,60,61^ even when presenting with early-stage HCC.^48,54^ These findings may be related but not limited to individual beliefs about liver cancer and treatment options (individual level of influence).
- **Impact of the biological domain**
  On the other hand, Asian American patients are more likely than other minority groups to receive resection^62^ and curative therapy in early-stage HCC.^48,54^ This may in part be explained by the higher rates of HBV-related HCC, including in noncirrhotic patients, within the Asian American community^63^ (community level of influence). As many patients with chronic HBV are recommended to undergo regular HCC surveillance irrespective of cirrhosis status,^19^ Asian American patients may be more likely to be diagnosed with early-stage or noncirrhotic HCC, both of which may be more amenable to curative treatments such as resection.^19^
- **Impact of the behavioral domain**
  Hispanic, Black, and Asian American patients are also less likely than White patients to receive liver transplantation for HCC.^60,64,65^ This difference is particularly important, as patients with HCC, if detected early, are usually the most likely to be listed for transplantation.^66^ One potential explanation for this finding could be that providers and transplant selection committees use varying definitions of “adequate social support,” which is a subjective eligibility criterion for transplant listing (interpersonal level of influence); for example, the intersection of race and SES might make it challenging for minority patients to have a caregiver who can afford to miss work while caring for a transplant recipient. Other reasons may include provider implicit bias affecting referral patterns^67^ and inconsistent thresholds of financial security^68^ that may impact the patient and provider interaction and prohibit transplant listing.
  These disparate findings also extend to end-of-life care; Hispanic and Black patients are again less likely than White patients to receive immunotherapy for advanced HCC^69^ and palliative care.^70^ Among Medicare beneficiaries, a greater proportion of racial and ethnic minority patients (44% vs. 31% of White patients) received no hospice care in their final year of life.^71^
  These findings suggest the importance of eliciting both patient and provider beliefs about liver cancer and treatment risks and benefits, to better understand the facilitators and barriers at the interpersonal level to delivering standard-of-care HCC treatments equitably across all racial and ethnic groups. The integration of community-engaged and qualitative research methods (eg, surveys, interviews, and focus groups) may be one way to systematically collect and report on these topics.

#### HCC-related mortality

- **Impact of the sociocultural environment domain**
  Individual SES (individual level of influence) can also impact HCC-related mortality. Patients with lower SES (vs. higher SES) experience higher HCC mortality irrespective of race and ethnicity; Black patients with low SES have the highest 5-year HCC mortality rate at 11.5%.^72^ Interestingly, this correlation with SES was not observed among AI/AN patients, but this may be related to the small sample size of AI/AN people included in the study. Such findings suggest that unmeasured or difficult to measure factors such as perceived social stigma, cultural identity, and financial stability may play important roles in the pathway toward HCC-related survival. Additional studies will need to better assess the relationship between these factors, including the possibility of other confounding factors such as individual health literacy and educational attainment and their impact on HCC-related mortality.
- **Impact of the health care system domain**
  Multiple studies have shown that an individual’s insurance status (individual level of influence) plays an important role in HCC care.^70,73–76^ First, minority patients are more likely than White patients to be uninsured or to receive public insurance (eg, Medicaid).^77,78^ In 2021, 18.8% and 47.4% of AI/AN, 17.7% and 37.7% of Hispanic, 9.6% and 45.3% of Black, 5.8% and 28.2% of Asian, and 5.7% and 35.7% of White Americans were uninsured and recipients of public insurance, respectively.^79^ Second, compared with Medicare or privately insured patients, Medicaid and uninsured patients with HCC have up to a 43% and 88% higher risk of death, respectively.^75,76^ In a multicenter study, lack of insurance was independently associated with decreased survival among patients with HCC.^74^ In another study, HCC mortality was not associated with race and ethnicity among White, Black, and Hispanic patients after adjusting for insurance coverage and health system.^73^ These findings suggest the importance of increasing individual access to health insurance to improve HCC-related mortality.
  On a larger scale, health policies that impact access to health insurance (societal level of influence) can also impact HCC outcomes. In a recent systematic review, Medicaid expansion has been associated with increased access to insurance coverage among patients with cancer and survivors overall and, in particular, among low-income people and minority people.^80^ Furthermore, within the US Veterans Affairs health system, where access to care is less of a concern, we found that generally observed disparities by race and ethnicity were not present, instead Black and Hispanic patients (vs. White) were more likely to receive HCC screening.^81^ These findings highlight some of the potentially modifiable factors within the health care system domain that currently influence HCC-related disparities. More studies are needed to evaluate the mechanism by which type of insurance coverage may be related to the timely receipt of HCC surveillance and/or access to HCC treatment, which may affect HCC-related mortality. Similarly, the impact of health policy changes on insurance coverage should be more closely assessed to better understand the societal level changes that could improve HCC-related survival both short term and long term.

### Proposed strategies to promote health equity in HCC care and research

The gaps in HCC incidence and mortality by race and ethnicity have remained stagnant over time.^82^ It is therefore important to urgently implement and disseminate effective interventions and develop strategies to engage under-represented patient populations to promote health equity in HCC care. Although a variety of interventions have previously aimed to improve outcomes along the HCC care continuum (Table 3), most of these studies have focused on the behavioral and health care system domains within the NIMHD framework, with interventions targeting downstream determinants of health, such as patient or provider behaviors in the clinical setting.^83–88^ Only 2 studies simultaneously targeted provider and patient or provider and community behaviors using reminder letters for HCC screening and educational presentations, respectively.^89,90^ Many of the studies sought to improve HCC screening rates, but only 2 of the studies were multicenter, limiting the generalizability of the results. Although 4 of the studies included a safety-net health system, all of these studies were also conducted in the same health system. Furthermore, studies rarely assessed the long-term impact of interventions, including its effects on HCC treatment and mortality. As HCC-related disparities are often driven by multiple factors, greater efforts are needed to diversify the study populations and the types of interventions used to identify the strategies that are most likely to be successful with broad dissemination.

**TABLE 3:** Selected articles on interventional studies focused on improving the HCC care continuum by framework domain

Framework domain	Author	Study population	Geographic location	Study design	Intervention	Results
1. Behavioral 2. Health care system	McMahon et al^90^	• Patients with chronic HBV (HBsAg + for 12 + months, N = 1487) residing in 126 Alaska Native villages • Intervention and control: • 100% Alaska Native	• Alaska	• Prospective 16-y cohort study • 11/1982-12/1998 • Historical control group (1969-10/1982)	• Reminder letter every 6 mo to obtain HCC screening using an AFP test • Letter was sent to all patients, village Community Health Aide, and regional health care provider	• Intervention group: • 32 cases of HCC, 22 patients treated with surgical resection • Patients with normal initial AFP level compared with control group: • 5-y tumor-free survival 29% (*p* = 0.004) • 10-y tumor-free survival 24% (*p* = 0.024) • Patients with HCC in intervention vs. control group: • 5-year survival 42% (*p* = 0.008) • 10-y survival 30% (*p* = 0.07)
1. Behavioral 2. Health care system	Beste et al^83^	• 8 VA facilities (Intervention-N = 790, Control-N = 2094) • Intervention: • 78.6% White • 10.1% Black • 3.4% Hispanic • 7.9% Other • 0.6% Unknown • Control: • 77.4% White • 1.9% Black • 2.6% Hispanic • 17.9% Other • 5.1% Unknown	• Tertiary care facility (Seattle, WA) • Primary care-focused facility (Tacoma, WA) • Regional liver transplant center (Portland, OR) • Lower-complexity facilities (Spokane and Walla Walla, WA; Boise, ID; Anchorage, AK; White City and Roseburg, OR)	• Quasi-experimental study • 1/2011-6/30/2012	• Computerized electronic health record reminder for HCC screening	• ↑ HCC screening by 51% in intervention facility (27.6%) vs. no change in control facilities (17.5%, *p* < .001) • ↑ HCC diagnosis (3.2% vs. 1.9%, *p* = 0.03)
1. Behavioral 2. Health care system	Mokdad et al^84^	• Parkland Health and Hospital System (Pre-N = 51, post-N = 45) • Preintervention: • 33% White • 33% Black • 24% Hispanic • 10% Asian • Postintervention: • 20% White • 38% Black • 29% Hispanic • 13% Asian	• Dallas County, TX	• Pre/postevaluation, single center • Pre: 2/2008-1/2010 • Post: 2/2010-1/2012	• Voice messaging system/electronic notification of HCC diagnosis to ordering and treatment physician	• ↓ median time from diagnosis to clinic (2.9 vs. 0.5 mo, *p* = 0.003) • ↓ median time from clinic to treatment (5.5 vs. 2.2 mo *p* = 0.005) • ↑ median survival (28.5 vs. 15.7 mo, *p* = 0.02)
1. Behavioral 2. Health care system	Singal et al^85^	• Parkland Health and Hospital System (N = 1800) • 28.3% White • 37.8% Hispanic • 32.1% Black • 1.7% Other/unknown	• Dallas County, TX	• Randomized clinical trial, single center • 12/2014-3/2016	• Mailed outreach + patient navigator vs. mailed outreach vs. usual care for HCC screening	• Over 6-month follow-up time: • ↑ HCC screening with mailed outreach + navigation (47.2%) vs. mailed outreach alone (44.5%) vs. usual care (24.3%)
1. Behavioral 2. Health care system	Singal et al^86^	• Parkland Health and Hospital System (N = 1800) • 28.3% White • 37.8% Hispanic • 32.1% Black • 1.7% Other/unknown	• Dallas County, TX	• Randomized clinical trial, single center • 12/2014-3/2017	• Mailed outreach + patient navigator vs. mailed outreach vs. usual care for HCC screening	• Over 18-month follow-up time: • ↑ HCC screening with mailed outreach + navigation (23.3%) vs. mailed outreach alone (17.8%) vs. usual care (7.3%)
1. Behavioral 2. Sociocultural 3. Health care system	Momin et al^89^	• Cherokee Nation Health Care Providers and Community Coalitions • Demographic information by race/ethnicity not provided	• Cherokee Nation	• Pre/postevaluation, single site • 8/2017-8/2018	• Didactic session for health care providers (15 min; 8 Project ECHO meetings) • Health care provider workshop (2-h; 7 clinics, 1 hospital) • Community coalition presentations (30 min; 5 meetings)	• ↑ in scores for HCC awareness, knowledge, ability, and intention after all 3 interventions
1. Behavioral 2. Health care system	Aby et al^88^	• VA Greater Los Angeles Health System (N = 129) • 39.5% White • 14.7% Black • 30.2% Hispanic • 1.6% Asian • 23.1% Other/Unknown	• Los Angeles, CA	• Pilot and feasibility quality improvement study • 1/2019-6/2019	• Phone outreach with a patient navigator	• Over 6-month follow-up time: • 32.5% reached by phone • 72.1% of those reached by phone completed HCC screening [adjusted OR 2.56 (1.03–6.33)]
1. Behavioral 2. Health care system	Singal et al^87^	• Parkland Health and Hospital System, UT Southwestern Medical Center, Michael E. DeBakey VA Medical Center (N = 2872) • Usual care: • 36.9% White • 32.6% Hispanic • 26.7% Black • 3.8% Other/Unknown • Outreach: • 37% White • 31.2% Hispanic • 28.5% Black • 3.2% Other/Unknown	• Dallas County, TX	• Randomized clinical trial, multicenter • 4/2018-12/2019	• Mailed outreach vs. usual care for HCC screening	• Over 12-month follow-up time: • ↑ semiannual HCC screening (35.1% s. 21.9%, *p* < .001) • ↓ no screening (29.8% vs. 43.5%, *p* < .001) • ↑ proportion of time covered by screening (41.3% vs. 31.0%, *p* < .001)

Abbreviations: AFP, alpha-fetoprotein test; HBV, hepatitis B virus; HCC, hepatocellular carcinoma; VA, Veterans Affairs.

**FIGURE 1:**
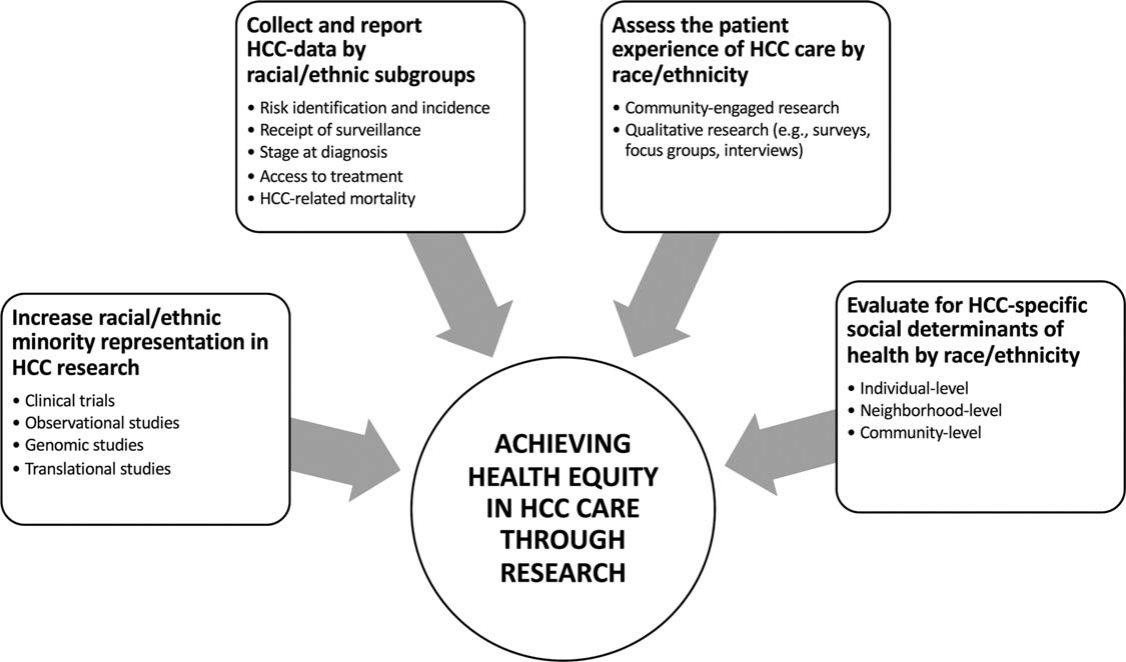
Proposed strategies to promote health equity in HCC care through research. Abbreviation: AI/AN, American Indian and Alaska Native.

We therefore propose the following strategies (Figure 1) to guide future HCC research that may more effectively address disparities in liver cancer care.

1. **Increase racial and ethnic minority representation in HCC research**. Greater efforts should be made to include racial and ethnic minority patients in both clinical and translational HCC research^91^ (eg, genomic studies evaluating the role of the *PNPLA3* gene polymorphism in NAFLD, survey studies assessing patient-reported outcomes after receipt of immunotherapy for advanced HCC, and clinical trials of biomarker development for HCC surveillance). In particular, there is a paucity of data from AI/AN, Native Hawaiian, and Pacific Islander communities, including information regarding gene polymorphism distribution, despite the high burden of HCC incidence and mortality in these communities. Building collaborations by investing in opportunities to grow clinical and research programs at nontertiary and nonacademic facilities (eg, community health centers, safety-net health systems, and Tribal Health Organizations), as racial and ethnic minority patients frequently receive care at these locations, will also help promote sustainable academic-community research relationships and thereby increase minority representation in HCC studies. As an example, the National Cancer Institute’s P20 grant mechanism has enabled us to establish a collaboration among the University of Washington, Fred Hutchinson Cancer Center, Alaska Native Tribal Health Consortium, and Cherokee Nation Health Services to study the use of biomarkers and abbreviated MRI-based HCC surveillance to promote early detection of HCC among AI/AN people.^92^ In indigenous communities, we have learned that collaborations that incorporate traditional knowledge can be particularly valuable for influencing upstream determinants of health.
2. **Collect and report data on HCC risk and incidence, surveillance, stage at diagnosis, treatment, and mortality by racial and ethnic subgroups**. HCC incidence and mortality rates differ by country of origin or subgroup, but studies often combine API, Hispanic, and Black patients into large racial and ethnic minority groups. Similarly, the AI/AN population includes many Tribal nations, each with its own distinct health system and community characteristics, which may have profound differences in cirrhosis—or HCC-related risk factors or outcomes. There is also a growing number of individuals who self-identify as a multiracial person. Thus, disaggregation and expansion of existing demographic variables are critical to enhancing the quality of data collection and interpretation in HCC research settings. Specifically, collecting data along the HCC care continuum by subgroup can be especially helpful when trying to assess factors within the physical/built environment, sociocultural, and health care system contexts, as specific subgroups of patients may have varying experiences or outcomes related to HCC care that would otherwise go unnoticed. For example, social stigma and beliefs related to HBV infection may differ by Asian American subgroups, which may affect future intervention targets.
3. **Assess the patient experience of HCC care within each racial and ethnic group**. At multiple steps along the HCC care continuum, the impact of patient-provider interactions, and their sociocultural environments cannot be overstated. While quantitative metrics related to HCC incidence and mortality are valuable, understanding the lived experiences of patients and providers can be instrumental in identifying key opportunities to address HCC-related disparities. In particular, because these experiences may vary by race and ethnicity, integrating community-based and mixed methods studies that include patient surveys, focus groups, and semistructured interviews will allow investigators to design interventions that are more likely to be successful within each minority group. Future studies would also benefit from including metrics on the implementation of an intervention (eg, acceptability, reach, adoption, cost, and sustainability) to evaluate and identify which approaches would be most successful within specific racial and ethnic minority groups.
4. **Evaluate for HCC-specific social determinants of health by race and ethnicity**. Studies have increasingly shown how HCC outcomes at the individual level are influenced by upstream factors such as neighborhood poverty, rurality of residence, and proximity to health care facilities. For example, among AI/AN people, HCC incidence varies by location and is highest in the Northern Plains, followed by Southwest, Pacific Coast, Southern Plains, East, and Alaska.^93^ However, the reasons for these differences are not clear. Thus, simultaneously collecting data on individual-level, neighborhood-level, and community-level characteristics among others may help explain the mechanism by which HCC disparities exist. In addition, evaluating the influence of socioeconomic characteristics and studying the interaction of clinical cofactors such as NAFLD and alcohol use between Tribal Groups may shed light on the variance of incidence rates. These findings can then inform the design and implementation of multilevel interventions, which target multiple factors involved in the HCC care pathway. Multilevel interventions are more likely to improve the impact and sustainability of future interventions.^94^

These 4 priorities to strategically improve the quality of data collection in HCC research will help inform the development of more successful programs and interventions that take into consideration the unique HCC-related experiences of each racial and ethnic group.

## CONCLUSIONS

HCC-related disparities by race and ethnicity exist both regionally and nationally in the United States. Observed differences along the HCC care continuum are related to many individual, interpersonal, community, and societal factors that span multiple spheres of influence including biological, behavioral, physical/built environment, sociocultural environment, and health care system domains. As we described in this narrative review, racial and ethnic disparities in HCC care are complex. As a result, we propose the use of an adapted NIMHD Research Framework to help organize and evaluate the intersection of factors that affect each step along the HCC care continuum to better address liver cancer disparities. We also present multiple strategies that may help improve the quality of future HCC studies as a way to enhance HCC care for racial and ethnic minority patients in the United States.
